# Editorial: Delineating the Visiting Experience: Matching Destination and Stakeholder Personalities

**DOI:** 10.3389/fpsyg.2020.01800

**Published:** 2020-08-06

**Authors:** Andreas Andronikidis, Victoria Bellou, Nikolaos Stylos, Chris A. Vassiliadis

**Affiliations:** ^1^Department of Business Administration, School of Business Administration, University of Macedonia, Thessaloniki, Greece; ^2^Department of Economics, University of Thessaly, Volos, Greece; ^3^Faculty of Social Sciences and Law, School of Economics Finance and Management, University of Bristol, Bristol, United Kingdom

**Keywords:** visiting experiences, tourism destinations, destination characteristics, stakeholder personalities, tourism industry

Research in tourism management has focused a lot on unraveling the “ideal” or “perfect” visiting experience, in order to support destination management organizations (DMOs), the hospitality industry and the local businesses decision-making process (Stylos and Vassiliadis, 2015; Stylos et al., 2016). This has been addressed from different viewpoints and across various managerial disciplines, i.e., destination branding, service experience, business strategy, and communication technologies (e.g., Mainolfi and Marino, 2018). Yet, given the fact that both destinations and stakeholders have their own personality, this “ideal” or “perfect” visiting experience, is rather utopian as it may significantly vary among individuals. In fact, research about destinations and stakeholders supports the idea that the “visiting experience” may vary, depending on the personality of each (Stienmetz, 2018). For this reason, this Research Topic seeks to delineate the role of personality of both destinations and stakeholders in achieving a match, which in turn would make the visiting experience a win-win situation for stakeholders.

Visitors are no longer prepared to do exactly what they are told and experience a destination exactly as others want them to. Furthermore, evolutions that have recently taken place in destination branding and the relevant novel tools that are available to both DMOs and visitors for promoting and exploring the destinations, respectively, have largely altered the relating matching of destination and stakeholders, as it was traditionally the case (Blazquez-Resino et al., 2016). In fact, listening to other visitors (with dissimilar profiles and personalities) could potentially improve their own visiting experience and vice-versa, for example via peer-to-peer (P2P) online communications (Bigné and Decrop, 2019).

Alternative matching mechanisms (e.g., p-e fit, push-pull, anticipation, place attachment among others) have been proposed to enlighten the decision-making process of stakeholders (e.g., visitors, DMOs) in a tourism destination context (Stylos et al., 2017; Bellou et al., 2018; Taylor and Norman, 2019). However, this is only in its infancy as there are many factors and matching parameters that could be included in relevant destination stakeholders' decision-making processes (Botti and Peypoch, 2013).

Thus, the editorial team put together a call for investigating innovative ways to delineate the visiting experience, and researchers have made three original research contributions to this Research Topic, which exemplify the contribution of different factors to matching destination and tourism stakeholder personalities.

The first study explores the extent to which individual preferences can be classified into clusters, thus offering a contemporary way of matching preferences with destinations and, subsequently, an optimal tourism destination planning. In doing so, Øgaard et al. have shown that the preference clusters identified, although offering useful insights, are non-sufficient when it comes to explaining the variability in certain aspects which are of primary interest during tourists' vacations. In fact, the various preference tourist segments demonstrated differences related to trip purchasing, or to the extent to which trip aspects were offered at the destination.

In the second study, Fotiadis et al. investigate a topic relating to organizational behavior in hospitality and the effect of work-life balance on employees' psychological well-being. The study indicates that psychological autonomy exerts positive effects on both psychological well-being and work-life balance, whereas psychological competence positively influences only psychological well-being. Nonetheless, psychological relatedness negatively affects both psychological well-being and work-life balance of hospitality employees, with potentially negative repercussions to the perceived experience in the eyes of hotel guests.

The third study examines the elements that make tourists' experiences interesting. Larsen et al. test two different theoretical frameworks via three surveys. In these three studies, novelty and familiarity are fundamental for tourists' experience, with tourists assessing the degree of interestingness of familiar and unfamiliar attractions, in surroundings they were familiar sand unfamiliar with, for themselves and other tourists. The authors conclude that tourists' experiences depend on a combination of familiarity and novelty, with findings suggesting this for both familiarity-, as well as for novelty-seekers.

Finally, the editorial team of this Research Topic offers a commentary on Blazquez-Resino et al. (2016) work, who empirically examined how the information posted by DMOs websites shape an individual's perceived destination image (PDI). The authors choose to focus on the cognitive and affective dimensions of image, positing that these two account for the tangible physical elements of the destinations under investigation, and the feelings aroused in visitors in response to internal or external stimuli by these destinations that can be rather easily be generated by the online context.

## Author Contributions

AA: conceptualization and draft write-up. VB: research approach and draft write-up. NS: conceptualization, draft write-up, and correspondence. CV: conceptualization and final review. All authors contributed to the article and approved the submitted version.

## Conflict of Interest

The authors declare that the research was conducted in the absence of any commercial or financial relationships that could be construed as a potential conflict of interest.
